# A Nation of Bastards? Registered Cohabitation, Childbearing, and First-Marriage Formation in Iceland, 1994–2013

**DOI:** 10.1007/s10680-020-09560-2

**Published:** 2020-04-07

**Authors:** Ari Klængur Jónsson

**Affiliations:** Department of Sociology, Stockholm University Demography Unit (SUDA), 106 91 Stockholm, Sweden

**Keywords:** Family formation, Cohabitation, Nonmarital childbearing, Marriage, Second demographic transition, Iceland

## Abstract

Nowhere in Europe is extramarital childbearing more pervasive than in Iceland. Roughly, 70% of children born in 2018 were conceived outside of marriage, thereof 83% of firstborn, which, on the surface, puts Iceland at the vanguard of a development often associated with a second demographic transition. In this study, we investigate the union formation behaviour of Icelandic women during a period of 20 years (1994–2013) with the objectives of gaining insight into the interplay of childbearing, registered cohabitation, and marriage and to enhance our understanding of the function of registered cohabitation in the family-building process. We use administrative population register data, covering the childbearing and marital history of the total female population born in Iceland during 1962–1997. The data are analysed by means of event history techniques and presented as annual indices of first-registered cohabitation and first-marriage formation, respectively. We find indications of forceful postponement of registering cohabitation over time, but a stable portion of around 80% of women registered cohabitation before any first marriage or age 46. Around 70% of women married before age 46, and the standardized marriage rates remained relatively stable during most of our study period. Our findings suggest that within a context such as the Icelandic one, most people tend to marry, regardless of the prevalence of cohabitation. We propose that registered cohabitation should be seen as providing a semi-regulated union status for prospective parents in relation to childbearing. Marriage on the other hand could be seen as providing an elevated union status to couples.

## Introduction

**Fig. 1:**
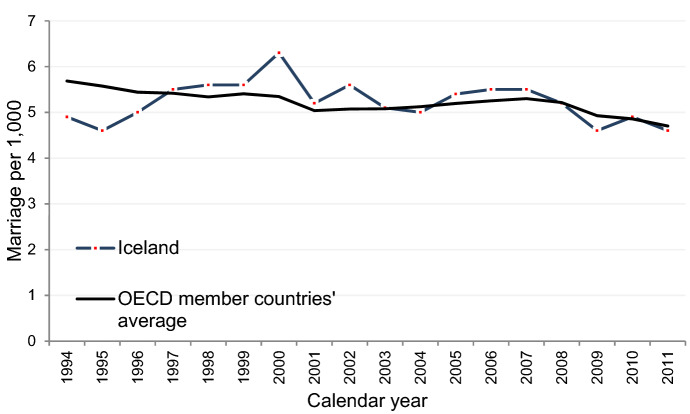
Number of marriages per 1000 of population 1994–2011. *Source*: OECD (2019b) (and author’s calculations). Note: Official figures pertaining to 2012 onwards for Iceland are not available

We have witnessed fundamental changes in family formation patterns in high-income countries during the past 50–60 years. These changes—often associated with a second demographic transition (SDT)—include lower marriage rates; increasing number of premarital births; and a higher prevalence of nonmarital cohabitation (Lesthaeghe 2010). Childbearing and marriage are becoming increasingly uncoupled. Iceland is among the vanguards in this development and, together with Chile and Costa Rica, the share of nonmarital births is larger than anywhere else among the countries covered by the OECD (OECD 2019a). According to recent data, seven out of ten Icelandic children are born to unwed mothers: 83% of firstborn; 67% of second-born; and 53% of third-born (Statistics Iceland 2019). The relatively high portion of premarital and nonmarital births has received international attention and raised questions about the future of marriage in Iceland (Weir 2017). It even earned the country the title of a Nation of Bastards in a religious American documentary (Faithful Word Baptist Church 2017; see also Farrow 2007). However, a quick glance over official statistics shows that the crude marriage rate of Iceland is on par with the OECD countries’ average (Fig. 1), which on the surface may appear counter-intuitive given the frontrunner status of the country in terms of the frequency of out-of-wedlock births.

This study context provides a strong case to investigate family formation from the perspective of the second demographic transition framework, discussed below, with respect to the country’s long history of cohabitation and its frontrunner status in terms of family change. Recent family demographic trends in Iceland might provide insight into future trends elsewhere. Despite the advanced development in the country, the (recent) family formation patterns have not been studied before. The objective of this study is to provide the first comprehensive overview of recent trends in union formation in Iceland. To that end, we display a system of annual indices of the propensity to register cohabitation and marry during a 20-year period: between 1994 and 2013. We rely on a proven method, established by Hoem (1991, 1993) and later elaborated by Andersson (1997, 1998, 1999), where we standardize union formation risks of age and other relevant demographic factors. We use population-based register data in our calculations, covering the total female population born in Iceland between 1962 and 1997. The data contain the childbearing and union histories of these women, including information about registered cohabitation. As our data stem from administrative population registers, we are restricted to couples that register their cohabitation. Registered cohabitation is a semi-formalized type of union that is associated with certain rights and obligations (discussed below). According to survey estimates, about three-in-four couples sharing a residence have registered their cohabitation (Jónsson 2019). Couples that do not register their union for one reason or another are not counted as cohabiters as we do not have any information on their actual family status. Nonetheless, with these reservations in mind, data on registered cohabitation give us a rare opportunity to study on a population level the prevalence of registered cohabitation over time to gain insight into the interplay of childbearing, registered cohabitation, and marriage and to enhance our understanding of the nature and function of registered cohabitation in the family-building process.

In what follows, we first provide a background of the general trends and potential determinants of changes in family formation in western countries. Subsequently, we turn our attention to the Icelandic context. After a discussion about the study background, we describe the data and methods and present the results. Finally, we conclude with a summary discussion.

## Background

Across Europe, marriage rates have declined substantially since the 1960s; fertility has decreased, in some places to very low levels; the number of unmarried couples has increased; and more children are born to unwed mothers (Sobotka and Toulemon 2008; Billari and Liefbroer 2010). An overview of EU28 statistics demonstrates that the Crude Marriage Rate decreased from 7.8 in 1965 to 4.1 in 2013, the Crude Divorce Rate increased from 0.8 to 1.9 during the same period, while the Crude Birth Rate of the EU28 countries dropped from 17.9 to 10.0 (Eurostat 2020). In terms of proportions of live births outside of marriage, the figure increased from around 20% in 1993 to 41% in 2013 (Eurostat 2020). Simultaneously, we observe increases in the median ages of the usual family-related indicators of the transition to adulthood: first-union formation, marriage, parenthood and to a lesser extent home leaving (Andersson and Philipov 2002; Andersson et al. 2017). On the surface, in most European countries this transformation appears in the form of a universal shift towards “less family” and more diverse living arrangements, fewer children—and a gradual retreat of marriage as the primary social institution for the family (Sobotka and Toulemon 2008; Esping-Andersen 2016).

One of the most prevalent theoretical frameworks in the demographic literature explaining this scenario is the second demographic transition thesis (SDT). According to the SDT—which has been used as somewhat of “a label, description, and even explanation for a plethora of diverse changes in fertility and family-related behaviours and attitudes” (Sobotka 2008, p. 172)—the family-related changes described above are, to describe it rather vaguely, a consequence of postmodern values. According to its proponents, the SDT is founded on an upsurge of higher-order needs, such as self-fulfilment, lifestyle, and personal development (van de Kaa 1996, 425). Childbearing becomes a choice rather than inevitability, competing with other “goods” in life, and is easily controlled by means of modern contraception. Simultaneously, we observe an increase in the frequency of extramarital births. While the two may appear contradictive, i.e. increased control over ones procreation and an expansion in the share of births out of wedlock, this contradiction is explained as a consequence of marriage ceasing to be the normative setting for childbearing (van de Kaa 1996). The strong normative structure, previously supported by both church and state, loses ground in an era governed by individual choice and freedom of self (Lesthaeghe 2010). Consequently, individuals make family-related choices based on their personal self-fulfilment (Carlson 2019). Cohabitation emerges as an alternative to marriage and can be viewed as one of the crucial components behind the de-standardization of traditional family formation patterns (Noack et al. 2013), and “the important engine driving the rise in nonmarital childbearing in Europe” (Kiernan 2004, 43).

Albeit the expansion of these SDT-related symptoms appears universal in Europe (Lesthaeghe 2010), highlighted by the increase in childbearing within cohabitating unions (Kiernan 2004; Perelli-Harris et al. 2009), there (still) is a shortage of evidence that indicates any convergence to a new general pattern of family demographic behaviour across developed countries (Sobotka and Toulemon 2008; Billari and Liefbroer 2010; Perelli-Harris and Sánchez Gassen 2012). While consistent country variations have been assigned to differentials in societal contexts (Lesthaeghe 2010; Carlson 2019; Heuvelline and Timberlake 2004; Thomson 2005; Billari and Liefbroer 2010; Perelli-Harris and Lyons-Amos 2015), they have equally brought about criticism of the SDT framework. Recent trends in family demography in the SDT-frontrunner states, the Nordic countries, demonstrate a turnaround towards increasing marriage rates since the late 1990s (Ohlsson-Wijk 2011) and a relatively high fertility in the region, in parallel to some of the highest nonmarital birth rates in Europe (Sobotka and Toulemon 2008), and persisting prevalence of nonmarital cohabitation (Andersson and Philipov 2002; Andersson et al. 2017). This development has nourished criticism of the SDT thesis to the extent that the credibility of the individualization perspective has been questioned and contributed to a hypothesis about a return to “more family” in the region (Esping-Andersen 2016, 9).

The relatively high gender egalitarianism in the Nordic countries has fuelled theories that instead make gender relations the explanatory point of departure of recent family changes (McDonald 2000a; Esping-Andersen and Billari 2015; Goldscheider et al. 2015). According to proponents of the gender relations framework, the impetus behind the ongoing development is thoroughly structural and to a much less extent based on ideation (Goldscheider et al. 2015). The phase of less family is considered as a consequence of imbalances between the expected societal roles of women on the one hand and their opportunities on the other (McDonald 2000a, b, 2013). Previous structural constraints stemming from the “traditional gender system” prevented women from being full participants in the labour market, as manifested by the breadwinner husband and stay-at-home wife (Goldscheider et al. 2015). Later, following improvements in women’s position outside the family and their expanded economic responsibilities, other kinds of structural constraints emerged—related to prolonged education and career building and manifested in postponed and even hindered childbearing and family formation (Goldscheider et al. 2015). During this period of “normative flux”, when women’s roles are advanced but society has yet to adapt in terms of new family norms and implementations of gender egalitarian family policies, it is argued that fertility tends to be low and union instability high (Esping-Andersen and Billari 2015). During the second phase of a “gender revolution”, gender equality will have increased and men will enter the private sphere with a feminization of men’s roles, relieving women of much of their caring responsibilities (Goldscheider et al. 2015). Consequently, the gender perspective expects greater ultimate family stability, including increased prevalence of marriage, and higher fertility (Goldscheider et al. 2015; Frejka et al. 2018).

Although the two theoretical frameworks originate from two distinct standpoints both sets of theories aim to explain the same overall developments, i.e. an initial trend towards less family highlighted by declining marriage intensities, increases in nonmarital childbearing, diffusion of cohabitation, and low(er) fertility. The gender perspective regards this development as an interim part during (the first phase of) a gender revolution while the SDT thesis approaches it as part of a transition, with a somewhat ambiguous endpoint. In terms of the Iceland’s stable childbearing trends and continuously high fertility, the development in this country appears to deviate somewhat from the expected pattern (cf. Jónsson 2017).

To a large extent, Iceland resembles the other Nordic countries—Denmark, Finland, Norway, and Sweden—in terms of its culture and institutional settings, and it shares the record high prevalence of cohabitation with the other Nordic countries (Kalmijn 2007; Andersson and Philipov 2002)—elaborated on below. The Icelandic culture is highly individualized when measured according to various cultural dimension indexes (Aðalsteinsson et al. 2011), and the country is also relatively secular: according to a survey, less than four in five Icelanders under the age of 25 said that they were religious, and none of the respondents in that age category believed that God created the Earth (Siðmennt 2016).

In relative terms, Iceland is highly gender egalitarian (World Economic Forum 2018), but there still is an imbalance in the levels of gender equity between “individual-oriented” and “family-oriented” institutions (cf. McDonald 2000b). Although Icelandic men occupy a world record share of father’s parental leave use, around one-third of the total leave days, women still exploit larger fraction of days (for a detailed discussion, see Jónsson 2018). According to survey estimates, Icelandic men spend about the same amount of time in household chores as men in the other Nordic countries, but which only amounts to about 70% of the time women spend on similar tasks (excluding childcare) (ISSP Research Group 2016, author’s calculations). Ever since the 1960s, female labour force participation in Iceland has been one of the highest in the world—despite that family policies were developed relatively late in Iceland compared to the other Nordic countries (Eydal 2008). Today, it is almost on par with that of men’s: about 80% of all women of childbearing ages are active in the labour market, but women are more likely than men to work part-time (Statistics Iceland 2019). During our study period (1994–2013), the number of women in tertiary education increased by about 150%, and women make currently roughly 60% of all university students (Statistics Iceland 2019). Notwithstanding, fertility has hardly declined to sub-replacement levels. The total fertility rate (TFR) has remained above the average in Europe, and it fluctuated around 2.1 ever since the mid-1970s and until the aftermath of the 2008 economic crisis. The projected completed fertility of the 1984 birth cohort is estimated at 2.3 children per woman (Statistics Iceland 2019). Iceland has thus never experienced the same drop in fertility as most other developed countries and as both sets of theories discussed above expect.

Our research questions are as follows: first, we set out to study if there have been any changes in the union formation behaviour in Iceland during the last few decades. If this is the case, we study the extent to which registered cohabitation has been replacing marriage. Based on the SDT narrative, one might expect an increase in the propensity to register cohabitation at the expense of the propensities to marry, whereas with regard to gender equality theory, we could expect to find an ultimate increase in marriage rates as Iceland has progressed further in terms of the second half of a gender revolution than most other developed countries. If registered cohabitation is viewed as an alternative to marriage, we might expect recent decreases in registered cohabitation due to increased commitment among couples which would be reflected in increasing marriage rates as the second half of the gender revolution progresses. However, if registered cohabitation is rather employed as an intermediate step in relation to childbearing and marriage, we can expect a more stable proportion of people registering cohabitation over time. Second, we ask whether there are indications of any specific period influences in family formation during our study period. Here, we are particularly interested in the last years of our study period. In 2008, Iceland suffered an economic breakdown when the country’s financial system collapsed. Unemployment increased sevenfold (Directorate of Labour 2015); the value of the Icelandic Krona dropped by some 50% (Einarsson et al. 2015); and social turmoil followed. In other contexts, poor labour market conditions have been negatively associated with union formation, both in the long-term with regard to marriage (Schaller 2013) and in terms of postponed and worse quality matching in couple formation in general (Ekert-Jaffe and Solaz 2001). Presumably, unemployment and low job security will reduce people’s confidence in the future, which might have deteriorating effects on couples’ commitment and their investment in family building (Ekert-Jaffe and Solaz 2001). Jónsson (2018) found that in the aftermath of the economic crisis fertility in Iceland declined, regardless of parity. If the crisis had any influence on union formation, we expect to find a drop in first-marriage intensities and the propensity to register cohabitation around the time when the crisis materialized, in tandem with the declining fertility.

## Cohabitation and Nonmarital Childbearing in Iceland

Iceland has a long tradition of nonmarital childbearing and cohabitation, which presumably dates back to the island’s settlement in the 800s and the “Old Norse culture” with its alleged high degree of gender equality and liberal attitudes towards premarital sex relations (Tomasson 1976, 256)—and thus long before even any first demographic transition took place. As far back as publication of official statistics go (mid-1800s in Iceland) the proportion of premarital births has been exceptionally high—even compared to “other liberal Scandinavian countries” (Björnsson 1969, 1). In the 1940s, one-in-four children was born to unwed mothers, as compared to a little more than the one-in-five children born out of wedlock in the 1870s (Trost 1978, 304). From the mid-1990s onwards over 60% of children in Iceland have been born outside of marriage (Statistics Iceland 2019).

Without going into detail about historical trends, the high rate of illegitimacy in Iceland in the nineteenth and early to mid-twentieth century is suggested to be a consequence of cohabitation without or prior to marriage—which again has been associated with the long-lasting effects of “*festar*” (Björnsson 1969), i.e. the mediaeval version of common law marriage, and the historical inefficacy of the Christian Church to preach the moral superiority of marriage in the country. In previous centuries, even the leading church men and bishops in Iceland had illigimate children without them having any social stigma attached to it (Gjerset 1925 as cited in Tomasson 1976, 258). Unmarried cohabitation and childbearing within this marriage-like union have thus, presumably, never been considered a deviant behaviour in Iceland (Björnsson 1969; Trost 1978). Nevertheless, even though nonmarital cohabitation has historical strong grounds in Iceland and is integrated into the culture of the country, with high tolerance towards nonmarital births attached to it, marriage was, at least during most of the twentieth century, still the final destination for the majority of the population. Cohabitation was mainly exercised as a prelude to marriage, during which time couples were establishing themselves economically—a behaviour that was not limited to the lower stratum of society (Björnsson 1969).

Apart from historical and cultural factors, the current frontrunner status of Iceland in terms of the prevalence of cohabitation and the high portion of premarital births can surely be traced to a progressive legislation concerning nonmarital cohabitation as an accepted family form during the past six to seven decades. To a large extent, regulations regarding nonmarital cohabitation in Iceland are similar to what they are in the other Nordic countries. In some matters, marriage and (registered) cohabitation are indistinguishable, but in others a clear legislative distinction is still made between the two unions (see below). The rationale for maintaining this distinction is twofold: first, to encourage couples to marry as it is considered the desirable *modus vivendi* by the legislative power; and second, to offer couples an alternative way to formalize their union (Alþingistíðindi 2000–2001). In general, those who are qualified to marry are also qualified to register cohabitation, e.g. in terms of age and interrelations. Registered cohabitation can be viewed as a light and less formalized version of the French PACS but contrary to PACS, Icelandic laws pertaining to registered cohabitation were until recently only intended for heterosexual couples (for a comparison see Waaldijk et al. 2017). In 2006, same-sex couples were also allowed to register their cohabitation (Alþingi 2006), and in 2010 the marriage act was reformed as well as to include same-sex couples (Alþingi 2010). Below we discuss the main similarities and differences between marriage and registered cohabitation in Iceland. For a comprehensive overview see Friðriksdóttir (2010, 2011); Alþingistíðindi 2000–2001; and Waaldijk et al. (2017).

### Regulations Concerning Registered Nonmarital Cohabitation in Iceland

Currently, there is no single legislation on registered cohabitation in Iceland (i.e. comparable to that of the marriage act (Alþingi 1993). Nevertheless, since the mid-twentieth century, various steps have been taken to enhance the rights of cohabiting couples—making this type of union more marriage-like. Already in 1943 nonmarital cohabitation is mentioned in Icelandic legislation as a part of the Act on Social Security, stipulating that cohabiting and married couples share the same obligations and rights in terms of national insurance. Since then legal provisions regarding nonmarital cohabitation have been implemented into various acts—usually next to clauses on marriage. Normally, stipulations regarding nonmarital cohabitation have a requirement about the union being registered; they depend on the length of the co-residence (usually one or two years), or they are conditional on couples having a child together (Alþingistíðindi 2000–2001).

In terms of social and family affairs, regulations pertaining to marriage usually apply to registered cohabiting couples as well. Child benefits and other family-related benefits are calculated the same way and are usually based on household income (Fjármálaráðuneytið 2004). Paternity is automatically recorded if couples have registered their cohabitation; biological cohabiting parents have the same rights in regard to parental leave as married couples, and upon union dissolution the general rule applies to registered cohabiting parents as to married parents: joint custody (Alþingi 2003a). Other couples need to go through more laborious administrative processes, even if they share a residence.

In terms of taxation, registered cohabitants can decide whether they are taxed as individuals or as married couples with a joint taxation and thus, depending on the partner’s income, being entitled to a tax discount. Married couples do not have this option and the spouses have the joint responsibility for each other’s taxes (Alþingi 2003b; Waaldijk et al. 2017). To a large extent, the same regulations still apply regardless whether couples have registered their cohabitation or married in regard to the Social Security Act (Alþingi 2007), i.e. in terms of social security benefits, family and maintenance benefits, and pension. Lone parents on the other hand are, in general, entitled to significantly more financial support than married couples and couples in registered cohabitation. In many cases they are also prioratized when it comes to childcare placements and entitled to reduced day care fees (Alþingistíðindi 2000–2001; Kristjánsson 2011). This modus operandi might sometimes act as an incentitive for couples not to register their cohabitation. However, couples sharing a residence are expected to register their cohabitation around the time of any childbirth, i.e. if they have not done so previously, and they are subjects to sanctions if they claim benefits for lone parents while sharing a residence at the same time (Alþingi 2003b).

With some simplification, the main differences between marriage and registered cohabitation in Iceland relate to statues regarding personal finances, in particular in relation to union dissolution and inheritance. There are no specific laws that pertain to financial matters of registered cohabiters or regulations that stipulate how couples shall split property upon union dissolution as there are for married couples. According to principle, each cohabitant is responsible for his or her properties and liabilities, and it is the individual who is the sole owner of what (s)he acquires during the course of the union.^1^ Married people are jointly liable in terms of tax and household debts, but there are no other financial liabilities unless there is a written agreement. Upon divorce, the principle of equal division of assets applies to married couples (Alþingistíðindi 2000–2001). There is no maintenance obligation between registered cohabiters during the course of the union as in marriage. However, both parents have maintenance obligation towards their children. There are also no general stipulations that encourage egalitarianism within a registered cohabiting union as there are for married couples in the marriage act (Alþingi 1993). Registered cohabitants can make a contract about joint financial affairs, which may include clauses about how to divide any property or debts upon separation (similar to a prenuptial agreement), but the validity of such contracts can be contested in court (Friðriksdóttir 2011). Alimony payments of any kind after union dissolution are not common practise in Iceland. Finally, contrary to married people, a registered cohabiting partner is not considered a legal heir, and the surviving partner does not have the right to retain an undivided estate. Unless there is a written testament, all properties go to whomever is considered next of kin according to law (Alþingistíðindi 2000–2001; Friðriksdóttir 2011).

## Data and Method

### Data

In our analysis, we rely on official population-based register data made available to us by Statistics Iceland. Everyone in Iceland is assigned a specific identification number used for, among other things, administrative purposes. By using an encrypted version of this identification number, we are able to merge individual-level information from different administrative population registers, which enables us to create full family-life histories of our subjects. This longitudinal data source gives us access to childbearing histories of the 1962–1997 birth cohorts of Icelandic-born women; their formal union histories (registered cohabitation and marriage); and the information we need for censoring purposes (migration history, age, and time of death).

Apart from a change that came into effect in 2006 and allowed same-sex couples to register their cohabitation (Alþingi 2006), and a reform in 2010 that extended the marriage act to include same-sex couples (Alþingi 2010), we are not aware of any significant changes that were made to the process of registration during the study period and which could have implications in the analysis. Registered same-sex cohabitation and marriages are included in our analyses from the date of change, which comprise around 1% of all registrations/marriages after the respective amendments came into effect (Statistics Iceland 2019).

The data source ensures that the data are accurate; we do not have to rely on the memory of our subjects; sampling errors are of no concern; and the population-based approach facilitates all statistical inference. A potential disadvantage of our data is that they are limited to demographic events (i.e. we do not have access to socio-economic variables that could be of interest), and we do not have information on men. Hence, we cannot detect if there are any differences in terms of, e.g. socio-economic status between those who register their cohabitation, those who do not register their union but share a residence, and those who marry. Also, we should keep in mind that the register data only contain official information. Informal cohabitation and other alternative living arrangements are not registered or covered in our dataset. We do not know when the actual cohabitation in terms of sharing a residence as a couple began, only when it was officially registered.

When constructing the union histories, we combined data from two registers: Registry of Change (Breytingarskrá) and the National Registry (Þjóðskrá). The first is our main data source as it includes information about the year and month of change in marital or registered cohabitation status, while the latter consists of yearly data only. We rely on the latter and assign June as the month of event occurrence if information is missing from the Registry of Change (less than 1% of all observations). We follow women from the age of 15 onward, until the exact age of 45 and 11 months, or 31 December 2013, whichever comes first. Our study population consists of 71,006 women who were born in Iceland during 1962–1997.

### Method

Our system of annual indices of first-(registered)-cohabitation risks and first-marriage risks, respectively, is based on an improved version of indirect standardization (for details, see Hoem 1991, 1993). We produce these indices by estimating piecewise constant exponential regression models—the risks of entering registered cohabitation and the risks of getting married—relative to the risks in the baseline year 2003, after we have standardized for various demographic factors: age, parity, and registered cohabitation status. (The last applies to first-marriage risks only.) This method enables us to investigate the underlying behaviour as it allows us to account for compositional changes in the study population over time that may influence union formation (Andersson 1998). For the analysis, we used the STATA software, version 15.1, and its built-in *st*-*commands*, which declare the data to be survival time data. See StataCorp. (2013) for detailed information and a list of relevant commands. For a comprehensive overview of event history analysis, see, e.g. Allison (1984), Blossfeld et al. (2007).

In all our main effects models, woman’s age is the main duration variable, categorized into single-year groups of ages 15–45, and parity is included as a time-varying covariate. In the marriage model, registered cohabitation status is included as a time-varying dummy, measuring whether women are living in registered cohabitation or not in any given month. All the variables are measured with a monthly accuracy. If registered cohabitation status and/or parity changes in the same month as any event of interest takes place, the new value is considered in the regression models. While our choice of baseline year is 2003, it is completely arbitrary as the general trends come out the same, regardless of base year. Our interpretations of the indices are comparable to those of a price index, demonstrating trends in prices relative to a given year (Andersson 1998)—hence, 2003 in our case. Women are right-censored at the time of any emigration after the age of 15, in the month of their 46th birthday or death, and in our registered cohabitation analysis at first marriage—whichever comes first.

As the estimated risks depend on both the exposure time and the number of registered events, any variation in our annual indices can reflect changes in either the timing of union formation, or the portion of women who enters a union (marriage or registered cohabitation) before age 46. To better distinguish between the two, we additionally present Kaplan–Meier descriptive synthetic cohort measures of the cumulative progressions to first-registered cohabitation and first marriage by age and calendar-year groups. In all the analyses, we study the two processes (first-registered cohabitation and first-marriage formation) independently of one another, with the exception that women are censored at first-marriage formation when estimating the risks of first-registered cohabitation (in cases where marriage precedes any first-registered cohabitation). The “exposure clock” is set to zero at age 15 and women enter the analyses in 1994 or the month they turn 15, whichever comes last. Hence, birth cohorts of women born before 1968 contribute full histories (until age 45 and 11 months), while the 1997 birth cohort only contributes to the last two calendar years of observation in 2012–2013. In all analyses, if women experience the event of interest prior to 1994 they are left-truncated. In what follows, the relative risks are interchangeably referred to as intensities, standardized rates, or propensities to register cohabitation or marry. Background statistics on the distribution of entries into union and exposure times under risk by variables and union type are available in Appendix Tables 2 and 3.

## Results

### First-Registered Cohabitation, First-Marriage Formation, and First Childbirth During 1994–2013

**Fig. 2:**
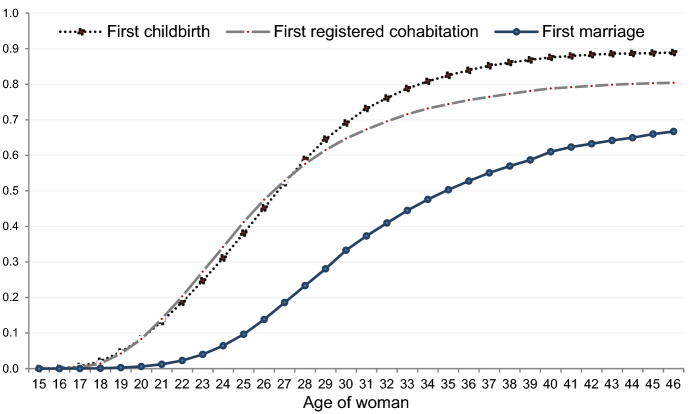
Kaplan–Meier cumulative probabilities of first childbirth, first-registered cohabitation, and first-marriage formation in Iceland, 1994–2013, by age of woman. *Source*: Icelandic register data, author’s calculations

Figure 2 presents Kaplan–Meier estimates of the cumulative probabilities of a synthetic cohort of Icelandic women to experience childbirth; registered cohabitation; and marriage during 1994–2013. The estimates indicate that around 90% of women become mothers before age 46. Jónsson (2017) has shown that there is no evidence of increased childlessness in Iceland during the past three to four decades (see also Appendix Fig. 5). With a potential risk of “aggregation fallacy”, i.e. the tendency to falsely infer standard sequences in real-life courses from median ages (Billari 2001, 124), Fig. 2 suggests that first childbearing and first formal cohabitation are closely associated: at age 28, 60% of women have given birth and the same portion has registered cohabitation, and the progressions to these two events, first-registered cohabitation and first birth, go hand in hand.

The cumulative probabilities indicate that three in every four women enter registered cohabitation as their first formal union, compared to one-in-ten women who marry without having registered any cohabitation first (see Appendix Fig. 6), but also, that the majority of first-registered cohabiting unions are later transformed into marriages. At age 30, one-in-three women have married according to our estimates, while more than twice as many have become a mother during our study period. At age 35, the portion of women who have married at least once has increased to 50%, and ten years later to almost 70% (Fig. 2). This indicates that while the association between first-marriage formation and first childbearing appears weak (demonstrated in more detail below), marriage is indeed part of the more advanced stages of the life course for the majority of women. Any claims about marriage being a critically endangered social institution in Iceland should therefore be treated with caution.

### Timing and Quantum in the Formation of First-Registered Cohabiting Union and First Marriage in Iceland, 1994–2013

**Fig. 3:**
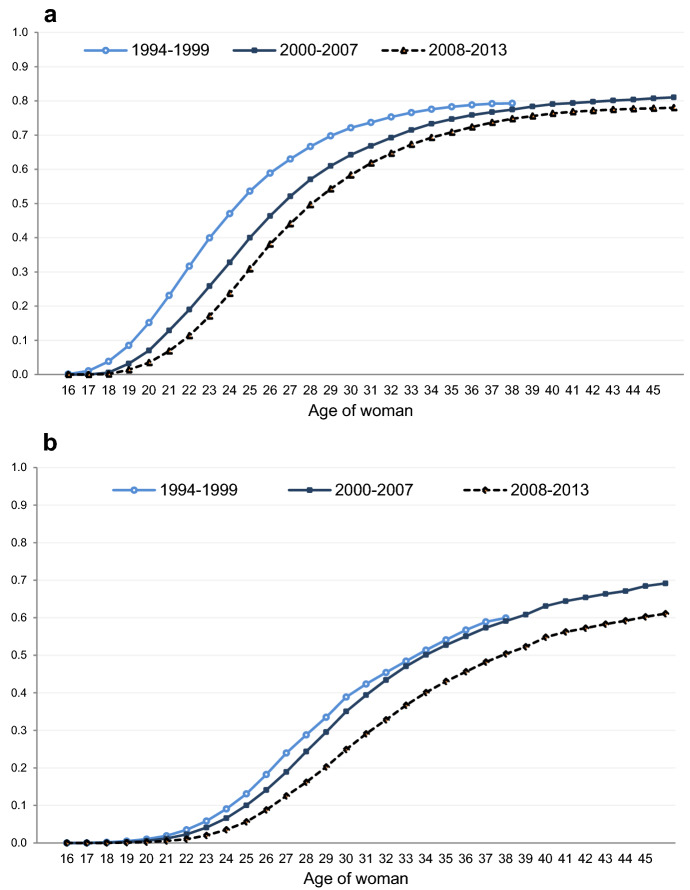
**a** First-registered cohabitation Kaplan–Meier cumulative probability estimates, 1994–2013, by age of woman over calendar-year periods in Iceland. *Source*: Icelandic register data, author’s calculations. **b** Kaplan–Meier cumulative probability estimates of first-marriage formation, 1994–2013, by age of woman over calendar-year periods in Iceland. *Source*: Icelandic register data, author’s calculations

**Fig. 4:**
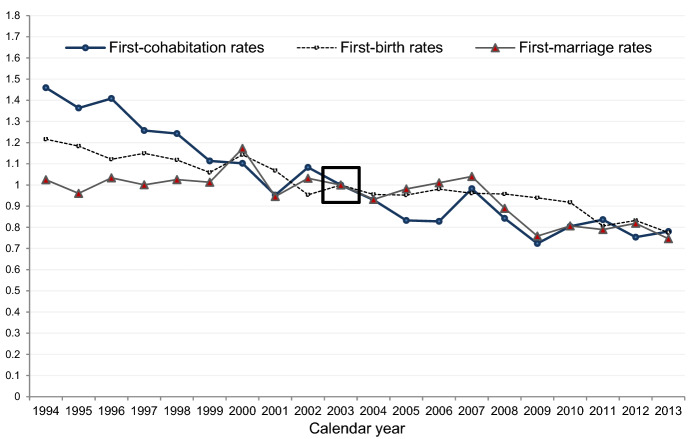
Relative risks of first-registered cohabitation, first-marriage formation, and first-birth fertility in Iceland 1994–2013. Standardized for age of woman (registered cohabitation status and parity). Rates are relative to the rates in 2003 for each event (separate models). *Source*: Icelandic register data, author’s calculations

Figure 3a presents Kaplan–Meier synthetic cohort estimates of the cumulative probability of Icelandic women that enter first-registered cohabitation before turning 46 or marrying directly by calendar-year period (1994–1999, 2000–2007, and 2008–2013).^2^ The estimates indicate a rather forceful postponement of first-registered cohabitation formation over time, but only minor changes in terms of the fractions that eventually register cohabitation.^3^ On average during 1994–2013, around 80% of women who do not marry directly register cohabitation before age 46, and this portion remained relatively stable during our study period. Over the 20 years, the age at which 50% of women had registered cohabitation, had, however, increased by almost four years: the age when half of the women had formally cohabited shifted from being around 24 years in 1994–1999 to 28 during 2008–2013. The observed postponement results in an overall decline in the propensity to register cohabitation over time—as shown by the relative risks presented in Fig. 4 below.

On the aggregate level, the observed increase in the age at which women register their first cohabitation appears to have had only marginal effects on the median age at first-marriage formation during the first two sub-periods we study, ranging from 1994 to 2007. Contrary to what we observed in Fig. 3a, the Kaplan–Meier synthetic cohort estimates of women entering marriage in Fig. 3b indicate that postponement of marriage was only minor during those initial years and that the age at which 50% of women had married was roughly the same (34 years) during 1994–1999 and 2000–2007, respectively. Also, the shape of the two curves does not demonstrate any retreat from marriage during 1994–2007 (with the reservation that ages 39–46 are missing from our first period).

A more palpable change occurs during the economic crisis period, i.e. during 2008–2013, which we did not detect to the same extent in the first cohabitation–formation patterns. During and in the aftermath of the crisis, we both observe a forceful postponement of marriage, and, at age 46, that only 60% of women would eventually marry compared to around 70% in the preceding periods (Fig. 3b). In order to better depict these changes in nuptiality behaviour over time, we next present standardized annual first-registered cohabitation and first-marriage formation rates.

### Period Trends in First-Registered Cohabitation and First-Marriage Formation

In Fig. 4, we display the annual indices of the propensity to register entry into first-registered cohabitation and first marriage, relative to the baseline year 2003. These standardized rates provide robust estimates of any behavioural changes over time as we control for compositional changes in the study population in terms of age, parity, and for marriage intensities, registered cohabitation status. For illustration purposes, we also present similar age-standardized rates of first-birth fertility (cf. Jónsson 2017).

As expected from previous accounts of postponed registered cohabitation patterns, we find a trend of declining propensity to register first cohabitation between 1994 and 2006 (Fig. 4), in tandem with declining first-birth intensities. We also observe a subsequent period of relatively stable risks of registering first cohabitation at the end of our study period. At the beginning of our period, women had roughly 70% higher risk of registering cohabitation than they had in 2006 and almost 90% higher risk than in 2013. However, as was already established in Fig. 3a, the declining propensity to register cohabitation is merely a consequence of postponement of first-registered cohabitation over time and does not reflect a retreat from this type of union. Further, more detailed estimates indicate a forceful decline in the propensity to register first cohabitation among women aged 15–26, but a parallel increase at older ages when models are estimated for these age groups, separately (see Appendix Fig. 7).

According to the standardized first-marriage rates in Fig. 4, the propensity to marry was relatively stable during 1994–2007. There are hardly any fluctuations in the rates between these calendar years, with the exception of a noticeable increase in year 2000, which is similar to millennium peaks observed in other Nordic countries (Ohlsson-Wijk 2011). Further analyses reveal minor changes in the age schedule of first-marriage formation during 1994–2007 (see Appendix Fig. 8), and little evidence of the gradient by parity changing over time. A small decline over time in the propensity to marry during 1994–2007 among childless and one-child mothers can be detected, and, similarly, a minor increase in marriage intensities among women at the higher parities (see Appendix Fig. 9).

Between 2007 and 2009, we observe a clear decrease in first-marriage rates (Fig. 4). The propensity to marry during and in the aftermath of the crisis is only around 70–80% of what it was prior to the economic crash. A further inspection of our estimates indicates that the drop between 2007 and 2009 was a result of declining marriage intensities across all our parameters—age, parity, and registered cohabitation status—and that the lower marriage risks were sustained in subsequent years by decreased propensity to marry among women under age 33, at parities 0 and 1, and by women who were living in registered cohabitation (see Appendix Figs. 8, 9, and 10, respectively). We conclude our battery of analyses with a closer look at the role of parity and registered cohabitation status (in marriage risks) in the union formation risks of Icelandic women.

On average during the period, one-child mothers have almost 90% higher intensities to register cohabitation than childless women (Table 1). This manifests that the official registration of the union is often associated with first childbirth. After that, the propensities to register cohabitation decrease with each additional child. Further analysis reveals a declining propensity over time to register cohabitation among childless women and among one-child mothers (see Appendix Fig. 11), which may suggest that unions are increasingly being registered post-birth rather than before becoming a mother. It is not inconceivable that family policies that enhance the rights of single parents, such as an income threshold for child benefits or admission qualifications for day care, might act as an incentive for couples to postpone union registration until later stages in the process of family building. For discussion of related patterns in other European countries, see Noack (2001), Knijn et al. (2007).

**Table 1:** Relative risks of first-registered cohabitation and of first-marriage formation in Iceland, 1994–2013, by parity and cohabitation status. Standardized for age of woman, and calendar year. *Source*: Icelandic register data, author’s calculations

	First-registered cohabitation		First-marriage formation	
	Haz. ratio	*P* > *z*	Haz. ratio	*P* > *z*
Parity 0	0.53***	0.000	0.86***	0.000
Parity 1	1	…	1	…
Parity 2	0.84***	0.000	1.30***	0.000
Parity 3	0.59***	0.000	1.36***	0.000
Parity 4	0.28***	0.000	1.36***	0.000
Parity 5	0.21**	0.029	1.67***	0.000
Not in cohabitation			1	…
In cohabitation			5.27***	0.000

**p < 0.05; ***p < 0.001

In general, and contrary to what we found regarding registered cohabitation, the propensity to marry increases with each additional child: on average, two- three-, and four-child mothers have around 30–40% higher risks of getting married compared to one-child mothers, and around 50–60% higher risks than childless women, after we have standardized for calendar year, age, and registered cohabitation status (Table 1). Further, women who live in registered cohabitation have on average a five-fold risk of marrying, compared to women not living in registered cohabitation. This underlines that registered cohabitation is the main family-building institution in Iceland, while marriage appears as more of a later-in-life venue of family change.

## Discussion

In terms of the second demographic transition narrative, one might have expected to find an increase in registered cohabitation over time, considering that it is a less committed and easier to dissolve union type than marriage—i.e. assuming that people would prefer to sustain more independence and relative freedom after committing to a union. However, counter to expectations, we do not observe any increase over time in the propensity to register cohabitation. The most palpable change in family formation that we observe during the 20-year long study period is that of postponed entry into this union type (and thus decreasing risk of first-registered cohabitation formation), which goes in parallel with a trend of delayed parenthood (see Jónsson 2017). Presumably, the lack of increases in registered cohabitation over time can be traced to the pervasiveness of this union type that already existed at the beginning of our study period. According to our estimates, about four in five women register cohabitation before turning 46, and this portion remained constant throughout the study period (1994–2013).

The prevalence of registered cohabitation is impressive, even compared to levels of informal cohabitation elsewhere in Northern Europe. The prevalence resembles those of less formal cohabitation in the other Nordic countries, Netherlands, Belgium, and France, where around 90% of women cohabit (informally) if they do not marry directly (Andersson et al. 2017, Table A-2b). Nonmarital cohabitation is integrated into Icelandic culture, and this family form is supported by legislation to the extent that almost no distinction is made between registered cohabitants and married couples in regard to childbearing and childrearing. In terms of Heuveline and Timberlake’s (2004) ideal types of cohabitation with respect to family formation, Iceland appears to represent a country such as Sweden where cohabitation is almost indistinguishable from marriage. Registered cohabitation enjoys general social acceptance and considerable institutional support; it does not seem to be viewed antithetical to marriage. Further, the majority of first-registered cohabitation episodes in Iceland are followed by the transition to marriage—unlike the French PACS that is rather considered an alternative to marriage (Heuveline and Timberlake 2004; Rault 2019).

When it comes to first-marriage formation the most interesting discovery is perhaps the stability in standardized first-marriage rates over time. We find scarce evidence of behavioural changes in first-marriage formation, and we had to wait for an economic collapse before observing any notable decline in standardized first-marriage rates. Also, contrary to what one might expect: the postponement observed over time in the formation of first-registered cohabitation did not lead to decreases in first-marriage intensities—at least when we focus on the decade-and-a-half long period prior to the economic crisis in 2008. The median age at first-marriage formation remained largely the same during 1994–2007. Hence, we find no evidence of any demise of marriage, and our findings may align better with the development as predicted by gender equality theory, than that of the second demographic transition thesis. As a consequence of increased individualism and associated value changes, the latter expects age at first-marriage formation to increase and long-term cohabitation to replace marriage—in tandem with sub-replacement fertility and plurality of living arrangements (Lasthaeghe 2010, 2014)—of which we find scarce evidence.

Our findings could be seen as an empirical reflection of the previous literature that suggests that family values in western countries have not changed as much as the individualization perspective predicts: in most countries, marriage is still considered an important institution, motherhood is still valued, and a vast majority of women aims for two or more children—with little changes observed in preferences over cohorts (Esping-Andersen 2016; Scott and Brown 2006). The driving force behind the development in Iceland thus appears to be related to structural rather than merely ideological factors—i.e. we observe evidence of postponement but nothing that suggests any significant quantum changes in family formation over time—which better corresponds to the core of arguments as presented by the proponents of gender theory (Goldscheider et al. 2015; Esping-Andersen 2016). As societies have progressed towards higher levels of gender equality, we might expect recuperation or persistent levels of different family-demographic outcomes. However, we do not necessarily expect the postponement of first-family formation that follows from prolonged education and high female labour force participation (Andersson 2000) to regress into earlier patterns.

Our results also suggest that marriage and registered cohabitation are motivated somewhat differently in Iceland. Registered cohabitation appears very child related as this family formation event appears to centre upon the arrival of children. This does not hold for marriage to the same extent. Within a context such as the Icelandic one, where the majority of children are born to unwed mothers and cohabitation has been semi-regulated, registered cohabitation should perhaps not be seen solely from the perspective of union formation. It should also be viewed from the perspective that registered cohabitation provides a semi-regulated status for prospective parents in relation to childbearing. Marriage on the other hand could be seen as providing an elevated union status to long-term couples.

Our findings harmonize with views that most people may want to marry, regardless of the diffusion of cohabitation within a country (Perelli-Harris and Sánches Gassen 2012). In case of union dissolution or the death of a spouse, married couples still have rights that exceed those of registered cohabitants, which may act as an incentive for some people to eventually marry.^4^ With increased age and responsibilities in terms of joint finances, housing (e.g. Holland 2012), and child support, it is likely that couples pay more attention to things such as reversionary rights and later-in-life security, which could act as a stimulator to get married, and thus partially explain why marriage has been as stable over time as our findings indicate. To a large extent, marriage may still be viewed as a symbolic declaration (Strandell 2017), or even a way to show of social status (Cherlin 2004).

Nevertheless, albeit marriage appears as a standard stage in family formation in Iceland, it is not a universal institution. According to our estimates a handsome portion of 30% of women had never married at age 45. At the onset of the economic crisis in 2008, marriage intensities declined quite sharply, and during 2009–2013 the propensity to marry was only 70–80% of what it was prior to the crisis. We can only offer suggestions as to why the marriage intensities declined as sharply as they did. Economic setback and increased unemployment are probable influencers (cf. Schaller 2013; Ekert-Jaffe and Solaz 2001), but additionally, the shock and insecurity that accompanied an economic crisis of this magnitude are likely compatriots. We can see that the crude marriage rates in other OECD countries declined as well during the economic crisis that hit most economies in 2008 (Fig. 1). However, it is likely that we will see recuperation in the Icelandic first-marriage rates in the years to come. The lower marriage intensities during the aftermath of the economic crisis were mainly sustained by lower marriage intensities of younger women at lower parities. Based on our findings from the pre-crisis period, these are groups of women who are still likely to marry.

As a concluding remark, our estimates indicate that the high nonmarital birth rate in Iceland is mainly a consequence of women having children before marriage, but it does not signal any retreat from marriage. Marriage still is a later-in-life event, and we find scarce evidence that the intensities to marry decline over time. Also, the vast majority of children appear to be born to stable couples but not to single women (see Appendix Fig. 5), an important distinction when analysing nonmarital childbearing (Thomson 2005). Most children will spend part of their childhood within the realm of marriage, but the extent to which this happens with two married biological parents and/or with a parent and step-parent awaits further analyses. In this study, we painted the family formation patterns with a broad brush in order to capture the main trends and development over time, at the expense of any other underlying trajectories and detailed sequences of events. The similarities in the developments over time of the transitions to first birth and first-registered cohabitation suggest that these two events are closely connected. It is plausible that the developments in the latter are mainly driven by the trends in Icelandic first-birth fertility. In other words, childless couples that presumably already share a residence seem prone to register their union around the time of childbirth. Future research, with different data, could investigate the interplay of informal cohabitation, the process of registering the union, and becoming a parent—which would provide an all-inclusive picture of Icelandic family formation patterns. Comparative analyses with similar countries with regard to the prevalence of marriage and premarital cohabitation, such as the other Nordic countries, Netherlands, Belgium, and France, would help further our understanding of the generalizability of our findings.

## Figures and Tables

**Fig. 5:**
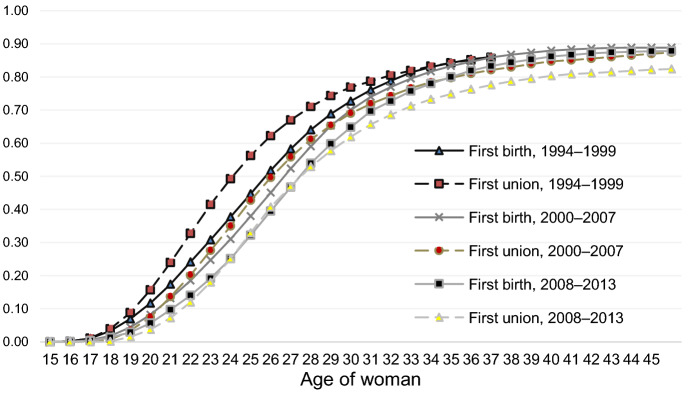
Kaplan–Meier cumulative probability estimates: First-registered union (registered cohabitation or marriage) and first birth 1994–2013, by age of woman over calendar-year periods in Iceland. *Source*: Icelandic register data, author’s calculations

**Fig. 6:**
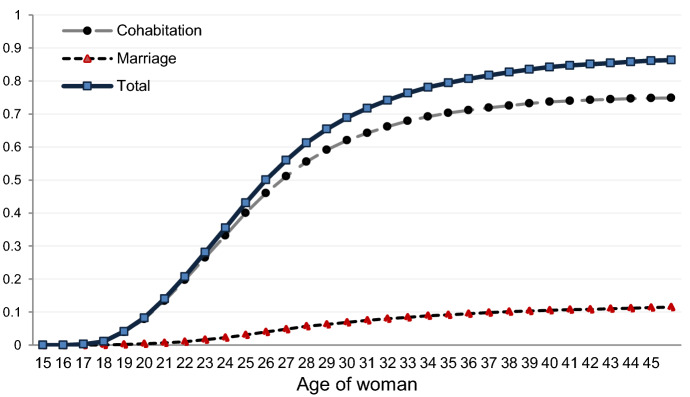
Cumulative incidence functions: Marriage versus cohabitation in Iceland 1994–2013, by age of woman. Competing-risks setup. *Source*: Icelandic register data, author’s calculations

**Fig. 7:**
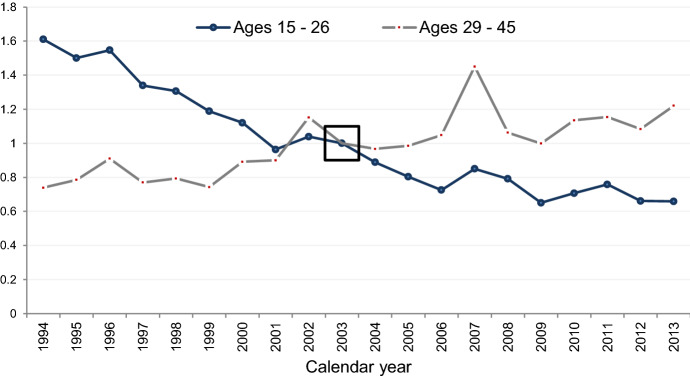
Relative risk of first-registered cohabitation in Iceland 1994–2013. Standardized for age of woman and parity, two age groups. Rates are relative to rates in 2003 for each age group (separate models). *Source*: Icelandic register data, author’s calculations

**Fig. 8:**
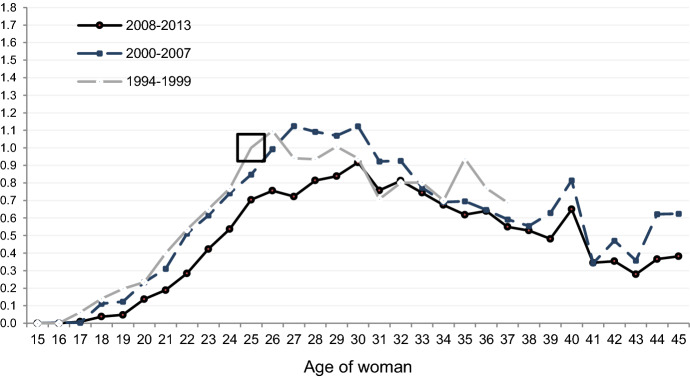
Relative risk of first-marriage formation in Iceland 1994–2013, by age. Standardized for parity and cohabitation status. Rates are relative to the rates of 25-year olds in 1994–1999. *Source*: Icelandic register data, author’s calculations

**Fig. 9:**
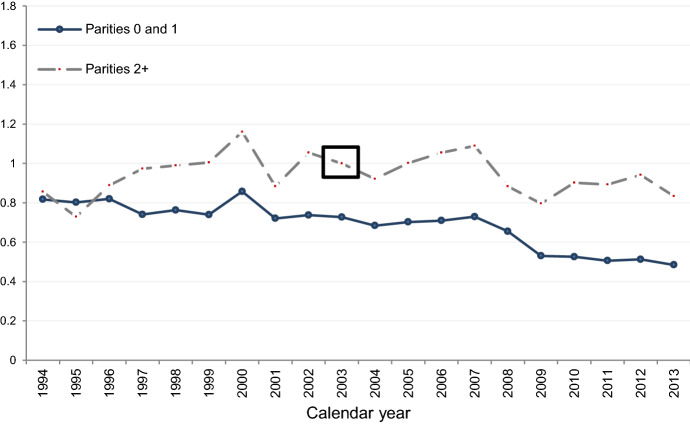
Relative risk of first-marriage formation in Iceland 1994–2013, by parity. Standardized for age and cohabitation status. Rates are relative to the rates of women in parity 2+ in 2003. *Source*: Icelandic register data, author’s calculations

**Fig. 10:**
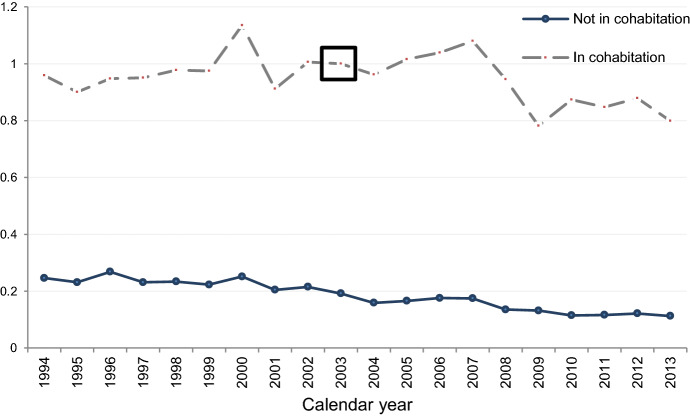
Relative risk of first-marriage formation in Iceland 1994–2013, by cohabitation status. Standardized for age and parity. Rates are relative to the rates of women in registered cohabitation in 2003. *Source*: Icelandic register data, author’s calculations

**Fig. 11:**
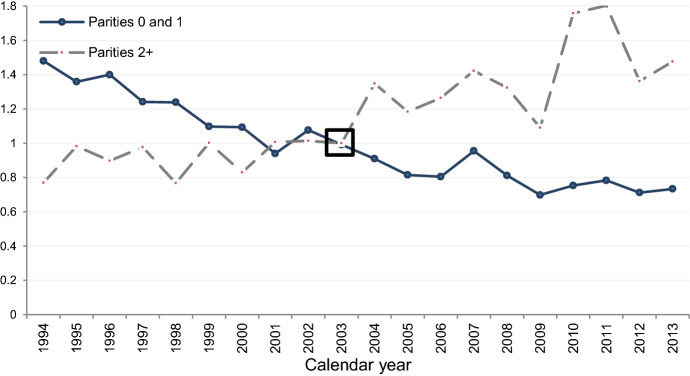
Relative risk of first-registered cohabitation in Iceland 1994–2013, by parity. Standardized for age. Rates are relative to the rates of women in parity 2+ in 2003. *Source*: Icelandic register data, author’s calculations

## Appendix

See Figs. 5, 6, 7, 8, 9, 10, and 11 and Tables 2 and 3.

**Table 2:** Background statistics: distribution of first-registered cohabitations and exposure times under risk by variables. *Source*: Icelandic register data, author’s calculations

Variable	Number of registrations	Woman-months under risk	%	Variables	Number of registrations	Woman-months under risk	%
*Age*				*Calendar year*			
15–18	1545	1,849,030	36.4	1994	1370	216,203	4.3
19	1536	437,536	8.6	1995	1284	220,495	4.3
20	2064	400,751	7.9	1996	1327	223,339	4.4
21	2260	356,060	7.0	1997	1194	225,748	4.4
22	2316	311,490	6.1	1998	1206	229,434	4.5
23	2167	265,405	5.2	1999	1104	233,819	4.6
24	2058	221,653	4.4	2000	1126	237,078	4.7
25	1719	182,940	3.6	2001	1009	240,410	4.7
26	1377	152,340	3.0	2002	1173	242,417	4.8
27	1136	127,717	2.5	2003	1094	245,663	4.8
28	877	108,474	2.1	2004	1039	251,563	4.9
29	687	94,094	1.9	2005	950	259,774	5.1
30	517	82,195	1.6	2006	973	269,092	5.3
31	432	72,943	1.4	2007	1172	274,266	5.4
32	374	63,866	1.3	2008	1020	279,037	5.5
33	261	54,369	1.1	2009	903	284,083	5.6
34	194	46,790	0.9	2010	1030	287,290	5.7
35	147	40,606	0.8	2011	1092	288,849	5.7
36	116	35,488	0.7	2012	1007	291,308	5.7
37	96	31,043	0.6	2013	1068	283,069	5.6
38	83	27,154	0.5				
39	62	23,970	0.5	*Parity*			
40	36	21,156	0.4	0	16,817	4,510,211	88.7
41	26	18,955	0.4	1	4,365	419,890	8.3
42	20	16,859	0.3	2	793	108,440	2.1
43	18	15,029	0.3	3	149	34,500	0.7
44	9	13,364	0.3	4	17	9,896	0.2
45	8	11,660	0.2				
No. of subjects: 71,006		No. of woman-months: 5,082,937			No. of registrations: 22,141

**Table 3:** Background statistics: distribution of first marriages and exposure times under risk by variables. *Source*: Icelandic register data, author’s calculations

Variables	Number of registrations	Woman-months under risk	%	Variables	Number of registrations	Woman-months under risk	%
*Age*				*Calendar year*			
15–18	100	1,865,509	24.7	1994	755	311,632	4.1
19	115	466,834	6.2	1995	763	323,345	4.3
20	214	453,570	6.0	1996	863	332,225	4.4
21	364	437,022	5.8	1997	865	339,987	4.5
22	602	418,847	5.5	1998	908	347,671	4.6
23	816	395,961	5.2	1999	917	355,390	4.7
24	1,040	370,432	4.9	2000	1,073	361,001	4.8
25	1,324	343,602	4.5	2001	869	365,744	4.8
26	1,486	317,418	4.2	2002	971	370,163	4.9
27	1,456	292,596	3.9	2003	954	375,622	5.0
28	1,422	268,217	3.5	2004	897	382,941	5.1
29	1,377	245,180	3.2	2005	958	391,788	5.2
30	1,306	221,421	2.9	2006	988	400,211	5.3
31	986	202,491	2.7	2007	1,025	406,246	5.4
32	956	182,077	2.4	2008	884	411,571	5.4
33	764	159,586	2.1	2009	748	414,937	5.5
34	603	140,092	1.9	2010	798	416,689	5.5
35	545	123,187	1.6	2011	788	417,957	5.5
36	454	109,284	1.4	2012	819	419,620	5.6
37	355	96,982	1.3	2013	750	411,048	5.4
38	293	85,462	1.1				
39	262	75,164	1.0	*Parity*			
40	288	64,926	0.9	0	4,415	5,014,921	66.4
41	116	56,874	0.8	1	5,352	1,334,544	17.7
42	115	49,768	0.7	2	5,516	830,938	11.0
43	76	43,706	0.6	3	1,910	300,063	4.0
44	88	37,709	0.5	4	400	75,322	1.0
45	70	31,871	0.4				
*Cohabitation status*							
Not in cohabitation	3,791	5,733,960	75.9				
In cohabitation	13,802	1,821,828	24.1				
No. of subjects: 71,006		No. of woman-months: 7,555,788			No. of marriages: 17,593		

## Footnotes

1 There are legal precedents where the courts have ruled that a registered cohabitant is entitled to a share of assets that have been accuired by the partner during the course of the union, albeit with consideration to the length of the cohabitation, any childbearing, and the financial solidarity of the couple (Friðriksdóttir 2011).

2 For similar Kaplan–Meier estimates of cumulative probabilities of childless Icelandic women progressing to parity one by calendar-year period see Appendix Fig. 5.

3 Our data are based on the registration of the event, hence we do not know if the postponement is restricted to the registration, or if women in general move in later with a partner.

4 It appears that a part of the population is not aware of the differences between marriage and registered cohabitation. In a survey conducted in Iceland in 2011, around 50% of the respondents wrongly believed that the same regulations applied to nonmarital cohabitation as to marriage as long as the couple had a child together and/or if the union was registered (Friðriksdóttir 2011).
